# Identifying medication errors in neonatal intensive care units: a two-center study

**DOI:** 10.1186/s12887-019-1748-4

**Published:** 2019-10-22

**Authors:** Kaveh Eslami, Fateme Aletayeb, Seyyed Mohammad Hassan Aletayeb, Leila Kouti, Amir Kamal Hardani

**Affiliations:** 1 Department of Clinical Pharmacy, Ahvaz Jundishapur University of Medical Sciences, Ahvaz, Iran; 2 Faculty of Pharmacy, Ahvaz Jundishapur University of Medical Sciences, Ahvaz, Iran; 3 Department of Pediatrics, Faculty of Medicine, Imam Khomeini Hospital, Ahvaz Jundishapur University of Medical Sciences, Ahvaz, Iran; 4 Student Research Committee, Ahvaz Jundishapur University of Medical Sciences, Ahvaz, IR Iran; 5 Department of Pediatrics, Faculty of Medicine, Abuzar Children’s Hospital, Ahvaz Jundishapur University of Medical Sciences, Ahvaz, Iran

**Keywords:** Neonate, Neonatal intensive care unit, Iran, Medication error

## Abstract

**Background:**

This study aimed to assess the types and frequency of medication errors in our NICUs (neonatal intensive care units).

**Methods:**

This descriptive cross-sectional study was conducted on two neonatal intensive care units of two hospitals over 3 months. Demographic information, drug information and total number of prescriptions for each neonate were extracted from medical records and assessed.

**Results:**

A total of 688 prescriptions for 44 types of drugs were checked for the assessment of medical records of 155 neonates. There were 509 medication errors, averaging (SD) 3.38 (+/− 5.49) errors per patient. Collectively, 116 neonates (74.8%) experienced at least one medication error. Term neonates and preterm neonates experienced 125 and 384 medication errors, respectively. The most frequent medication errors were wrong dosage by physicians in prescription phase [WU1] (142 errors; 28%) and not administering medication by nurse in administration phase (146 errors; 29%). Of total 688 prescriptions, 127 errors were recorded. In this regard, lack of time and/or date of order were the most common errors.

**Conclusions:**

The most frequent medication errors were wrong dosage and not administering the medication to patient, and on the quality of prescribing, lack of time and/or date of order was the most frequent one. Medication errors happened more frequently in preterm neonates (*P* < 0.001). We think that using computerized physician order entry (CPOE) system and increasing the nurse-to-patient ratio can reduce the possibility of medication errors.

## Introduction

Medical error refers to all errors occurring in health care systems such as equipment failure, diagnostic–therapeutic errors and medication errors. Medication errors are one of the most important and preventable type of medical errors and defined as any preventable event which may result in the misuse of drugs or patient injury [1, 2].

Studies show that patients with complex clinical conditions who receive multiple medications and interventions are at greater risk of experiencing medication errors [3].

Neonates are at risk of medication errors once they are admitted to intensive care units because they undergo invasive and non-invasive diagnostic–therapeutic procedure as well as receiving multiple drugs for their recovery and survival.

Prescribing medications based on birth weight, gestational age, postnatal age, and also their premature systems of absorption, metabolism, and secretion of drugs lead to dramatic changes in drug dosage and interval range in this population. These conditions necessitate performing dilutions for appropriate dosage for each neonate, thus leading to medication errors. Studies have shown that the risk of medication errors in neonatal wards is eight times more likely than that of adults in the hospital setting [4].

Available studies indicate that 13–91 medication errors per 100 admissions may happen in the NICU [5, 6]. Several studies conducted in developed countries showed that medication error mostly occurred during prescribing phase [4, 6–8]. The most common types of medication errors are drug dosage error, frequency, and route of administration [9–11]. The most effective practical steps, to reduce medication errors, included preparing pre-designed medical order sheet and the presence of a clinical pharmacist when visiting neonates as well as training doctors and nurses [11]. As no studies have been done on medication errors in NICUs at southwest of Iran, the present study was done to determine the type and frequency of medication errors, identify risk factors and take practical steps to reduce medication errors.

## Materials and methods

### Study setting

This descriptive cross-sectional study was performed in two NICUs at Abuzar and Imam Khomeini hospitals in Ahvaz, southwest of Iran. Abuzar is a pediatric academic hospital with a 15 bed NICU. Two attending neonatologists visited all neonates every day. Patients are referred from other centers to this NICU (outborn) and include term and preterm infants with over or at 32 weeks gestational age, mostly greater than a birthweight of 1500 g. All admitted patient are term and preterm infants more than 32 weeks gestation, weighing more than 1.5 kg, and transferred from another center The NICU at Imam Khomeini Hospital is a level 3 NICU with 45 beds, located in a general educational and medical hospital where almost all hospitalized neonates are born in the same hospital (inborn). Three attending neonatologists in this ward visited patients every day. In the NICU of Imam Khomeini hospital, most neonates are preterm with gestational age between 28 and 36 weeks. There are only a limited number of extremely premature neonates (< 28 weeks). Considering the birth weight, Most admitted patients are low birth weight or very low birth weight. Few admissions are extremely low birth weight.

### Population

Neonates were admitted to two NICUs during a 3-month period between January and March 2016. We included infants admitted for 24 h or more and received at least one therapeutic drug. Neonates were divided into three categories by birth weight (≥ 2.5 kg or < 2.5 kg), gestational age (≥ 37 weeks) or preterm (< 37 weeks) and length of stay (≤ 7 days or > 7 days).

### Data collection

We collected data on neonates and their medications. Neonatal demographic characteristics included sex, gestational age, birth weight, weight at time of admission, postnatal age, and hospital length of stay. Information of medications extracted from the medical order sheets of the admitted neonates included the names of drugs, dosage forms, drug dosage, route of administration, frequency, the number of drugs received, the number of doses received, and total number of prescriptions for each patient. Factors such as parenteral nutrition, topical medications, serums and electrolytes, oxygen therapy, blood products, vaccines, vitamins, and contrasts excluded from the study. The researcher collected all the information about the neonates from admission to discharge or death. Medication errors detected from the extracted information were merely recorded but their development was not interpreted.

Orders were manually written by residents (in the morning shifts under the supervision of attending physician, and in the afternoon and night shifts by the senior residents) and were transcribed to the Kardex by nursing staff. (Kardex is a card-filing system that allows quick reference to the particular needs of each patient for certain aspects of nursing care. Included on the card may be a schedule of medications, diet, any special problems, a schedule of treatments and procedures, and a care plan. The Kardex is updated as necessary and is usually kept at the nurses’ station.).

### Medication errors

In this study, medication errors were evaluated based on the definition of the National Coordinating Council for Medication Error Reporting and Prevention (NCCMERP), an independent association with 22 national organizations. Medication error is defined as any preventable event that may cause medication misuse or harm while the medication is under the control of the health care professional, patient, or consumer [2]. The medication errors include dispensing, prescription, transcription, preparation, and administration errors.

The present study reviewed prescription and administration errors.

### Prescription errors

In the present study, prescription errors are defined as wrong dosage, route of administration, and intervals. Wrong dosage was considered as a deviation of ≥10% from the references. Two sources such as the 22nd edition of Lexicomp, the Pediatric and Neonatal Dosage Handbook, and the 20th edition of the online reference Harriet Lane were used as reference [12, 13].

### Administration errors

Administration errors included failing to administer medication (the nurse would not administer the medication prescribed by the doctor for the patient till the end of the shift), administering the wrong medication to patients, administering medication that has not been prescribed by physician administering medication to the wrong patient, failing to match the dosage given to the patient with the dosage prescribed by the physician, failing to match the route of administration with the physician ’s order and, failing to match dosing intervals with the physician’s order and administering the medication after its discontinuation by the physician.

Another aim of the present study was to investigate the quality of the prescriptions.

In this section, seven aspects were assessed for each drug prescription. They included illegible prescriptions, unspecified dosage form, unspecified and/or vague measurement unit for the medication, unspecified route of administration, unspecified drug intervals, failing to record the time and/or the date of the order and failing to record the specifications of issuer including their name and /or signature.

The code of ethics (IR.AJUMS.REC.1395.96) were issued by the Ethics Committee of Ahvaz Jundishapur University of Medical Sciences. Results were analyzed statistically after extracting the intended information from the neonatal records.

### Statistical analysis

The data were described using the mean and standard deviation, median and interquartile range, and analyzed using t-test (in case of the assumption of normality of the data), Mann–Whitney (Median) (in the absence of the assumption of normality of the data), Fisher’s exact test, Chi-square and Wilcoxon tests and a Logistic regression analysis. For logistic regression, a multivariable regression model was used to investigate the effects of neonates’ characteristics on prescription errors independently. All analyses were performed using SPSS 20.

## Results

Of 155 examined cases, 102 (65.8%) were male, 76 (49%) were preterm, 71 (45.8%) were low-birth weight neonates, 100 (64.5%) were outburn neonates and 141 (91%) discharged alive. One hundred fifty-five patients were admitted for 1579 days and the mean of hospital length of stay was 10.19 ± 11.77 days for each neonate (min: 1; max: 65). The average birth weight was 2.54 ± 0.98 kg (min: 0.44; max: 4.5).

A total of 688 prescriptions were written for the studied patients, and the mean of prescriptions was 4.44 ± 3.21 for each patient; 7629 drug dosages and 44 types of drugs were prescribed.

There were 509 medication errors (M = 3.28 ± 5.49 for each patient). Of 155 patients, 116 neonates (74.8%) experienced at least one error. Prescription errors (*n* = 289), administration errors (*n* = 220), and medication errors in term neonates (*n* = 125) and preterm neonates (*n* = 384) had been occurred (*P* < 0.01).

The neonates were divided into two groups based on the hospital length of stay (admission of ≥7 days and of < 7 days). There was a significant difference between hospital length of stay and the frequency of medication errors (*P* < 0.001). The number of errors in these two groups was 128 and 381; respectively.

The frequency of errors in neonates weighing less than 2500 g (2.5 kg) at birth and those with normal birth weight (≥ 2.5 kg) was 373 and 136, respectively (*P* < 0.001).

Table 1 displays demographic information for the neonates and the frequency of medication errors, prescriptions, drugs and doses. .

**Table 1:** Comparison of medication errors, prescriptions, drugs and doses between term and preterm neonates

Neonates characteristics	N (%)	Frequency of medication errors/100 prescription (%)		*P*-value	Prescriptions^#^ (Median (IQR))	*P*-value	Drugs^#^ (Median (IQR))	*P*-value	Doses^#^ (Median (IQR))	*P*-value
		Found	Not found							
Sex				< 0.0001^##^				0.79		0.59
Male	102 (65.8)	65.6	34.4		4 (2–5.25)	0.9	3 (2–5)		27.5 (5–16.7)	
Female	53 (34.2)	91.8	8.2		3 (2–6)		3 (2–5)			
Gestational age (weeks)				< 0.0001^##^		0.019^##^		0.02^##^		0.93
Term (≥37)	79 (51)	41.4	58.6		3 (2–5)		3 (2–4)		28 (17–59)	
Preterm (< 37)	76 (49)	99.5	0.5		4 (2–7)		4 (2–5)		33 (15–79)	
Birth weight (kg)				< 0.0001^##^		0.008^##^		0.017^##^		0.94
Normal (≥2.5)	84 (54.24)	42.8	57.2		3 (2–5)		3 (2–4)		28.5 (15.25–57.5)	
Low birth weight (< 2.5)	71 (5.8)	100	0		4 (2–7)		4 (2–6)		37 (16–82)	
Hospital length of stay (days)				< 0.0001^##^		0.98		< 0.91		0.064
> 7	63 (40)	40.3	59.7		3 (2–4)		3 (2–5)		42 (19–76.5)	
≤ 7	93 (60)	100	0		5 (3–8)		3 (2–5)		23 (15–52)	
Age at admission (days)				< 0.0001^##^		< 0.0001^##^		< 0.0001^##^		< 0.0001^##^
≥ 7	68 (43.9)	52	48		4 (2–5.75)		2 (2–4)		19 (12–37)	
< 7	78 (56.1)	91.1	8.9		3 (2–6)		4 (3–6)		66.5 (37–106.25)	

# Non-normally distributed data, expressed as median and interquartile range

## *P*-value < 0.05 indicates statistically significant difference

The most common errors included administering wrong dosage (*n* = 142, 28%) and failing to administer the medication by nurse (*n* = 146, 29%). The frequency of wrong route of administration and wrong intervals was 43 (8.4%) and 104 errors (20.4%), respectively. On the other hand, some other errors included s administering medication that has not been prescribed by physician (*n* = 2, (0.3%)), failing to match the dosage given to the patient with the physician’s order (*n* = 38, (7.4%)), failing to match the route of administration with the physician’s order (*n* = 3, 0.5%), failing to match the intervals with the physician’s order (*n* = 27, 5.3%), and administering medication after its discontinuation by the physician (*n* = 4, 0.7%).

There were no errors in the following variables: administering the wrong medication to patients, administering medication to the wrong patient, and mismatching the dosage form with the physician’s order.

In the prescribing phase, the wrong dosage error was significantly higher in preterm neonates than term neonates (*P* < 0.005), and hospital length of stay was associated with wrong route of administration and wrong dosing intervals (*P* < 0.014 and *P* < 0.007, respectively) (Table 2).

**Table 2:** The effect of neonates’ characteristics on different types of prescription errors, a multivariable regression model

	OR	95%CI^a^	*P*-value
Wrong dose			
Birth weight (kg) (< 2.5 to ≥2.5)	0.51	(0.5–4.43)	0.54
Gestational age (weeks) (< 37 to ≥37)	21.62	(2.52–184.91)	0.005**
Hospital length of stay (days) (< 7 to ≥7)	2.18	(0.94–5.07)	0.06
Sex (Female to male)	1.55	(0.67–3.57)	0.3
Wrong route of administration			
Birth weight (kg) (< 2.5 to ≥2.5)	1.48	(0.26–8.32)	0.65
Gestational age (weeks) (< 37 to ≥37)	0.92	(0.16–5.39)	0.93
Hospital length of stay (days) (< 7 to ≥7)	2.81	(1.23–6.39)	0.014**
Sex (Female to male)	1.11	(0.49–2.49)	0.79
Wrong dosing interval			
Birth weight (kg) (< 2.5 to ≥2.5)	3.7	(0.87–15.63)	0.07
Gestational age (weeks) (< 37 to ≥37)	2.23	(0.52–9.33)	0.27
Hospital length of stay (days) (< 7 to ≥7)	2.94	(1.34–6.43)	0.007**
Sex (Female to male)	1.41	(0.63–3.13)	0.39

** *P*-value < 005 indicates statistically significant difference

^a^ 95% CI: 95% Confidence interval

Considering the quality of prescribing, 127 errors were recorded in 688 prescriptions (18.4%); of which unspecified time and/or date of order (58%) was the most common. Illegible prescriptions (1.5%), unspecified or dubious drug dosage units (5.5%), unspecified drug dosage form (1%), unspecified route of administration (30%), unspecified dosing intervals (3%), and the absence of the name and/or signature of the issuer (1%) were other errors regarding to the quality of prescribing.

Among 33 drugs involved in medication errors, errors (53.8%) mostly occurred in antibiotics group, and intravenous injection was the most commonly reported method (70%) in terms of the route of administration. Most commonly prescribed drugs were ampicillin, gentamicin, vancomycin, cefotaxime and salbutamol. Gentamicin, ampicillin, caffeine, ranitidine, and vancomycin were ordered hierarchically in terms of frequency of medication errors.

## Discussion

The results of the present study revealed that 74.8% of patients, in two NICUs in Iran, had at least one or more medication errors. This error rate was similarly reported by Truter as 78% [14]. Since about 3/4 of the hospitalized neonates have experienced medication errors, effective interventions are needed. This great number of medication error may be because of considering many variables as medication errors in our study compared to the others. In the present study, 12 variables were considered as medication errors. In a study of Lerner et al. in Brazil [15], 54% of patients experienced an error which was less than that of our study. Differences in the frequency of reported medication errors can be due to different definitions of medication error and methodologies which prevents accurate comparison.

Of total 155 patient charts reviewed with 688 prescriptions, 289 prescribing errors (42%) occurred. Pallas et al. [8] reported 39.5%. prescribing error In Truter et al.’s study [14], the frequency of medication error in the prescribing phase was reported 47%, which is consistent with that of the present study. In a recent review by Krzyzaniak, the prescription error rate was 14–74%. The reason for high amount of prescription errors can be the insufficient experience of physicians, highly populated in-patient wards, heavy workload, manual prescriptions, the need for repeated numerical computations to determine the dosage and individual prescriptions for each neonate.

The frequency of failing to administer the medication by nurses was 29%, which indicates the relatively high rate of this type of error. Frequency of the mentioned administration error in F. Nawwar et al.’s study [16] in two NICUs was reported to be 3.7 and 3.4%; respectively which is lower than that of our study. The reason for such a high frequent error for failing to administer the medication can be the number of infants undertaken by nurses in NICUs (8–10 infants for one nurse) which is far from the global defined standards. Further, the reason for this wide difference is due to the different feature of the present study based on chart review and the observational nature of the methodology used by F. Nawwar et al. In the present study, the nurses who were responsible to administer medicine to the neonates forgot to do it or register the facts and figures in the neonates’ records due to their high workload.

In many studies, the most common medication error was the dosage error and reported to be 42 and 51.5% in two different studies [1, 17]. Based on the present study, the frequency of this error was 28%, which is clearly lower than of previous studies which can be due to the frequent control of drug dosage when prescribing, and saving the main sources of drug information in resident’s cell phones.

In the present study, there was no error in giving medication to the wrong patient. However, Dabliz and Levine’s study [18] estimated that about 25% of medication errors arise from giving medication to the wrong patient. The lack of this error may be due to nurses’ failure to record the events or the presence of numerous identity evidence for the neonates such as the baby’s bed and identity ring on the wrist or ankle for all neonates.

The present study showed that 57 and 43% of medication errors were made by doctors and nurses respectively; as opposed to a study by Shane Pawluk in which 98.5 and 1.5% of errors were made by doctors and nurses, respectively [19]. According to Pawluk, this low administration errors by nurses differed widely from the results of our study and the review study of Krzyzaniak [20], who reported 31–63% administration errors in various studies. The reasons for such a great number of administration errors in the present study can be attributed to the various variables related to nurses’ errors. In addition, inadequate number of nurses available in the ward, frequent and long shifts followed by prolonged sleep deprivations, and poor nursing education can be factors leading to increasing the number of mistakes made by nurses.

The incidence of medication errors in preterm neonates admitted to intensive care units was significantly higher than that of term neonates (75.4% versus 24.6%). The findings of Machado et al. [21] indicates the same results. The possibility of a medication error is due to a longer period of hospital stay of premature neonates in the NICU and receiving more drugs with smaller dosages, the need for more accurate and frequent calculations, the necessity of changing the dosage and dosing intervals in the long-term hospital stay. In this regard, prematurity is principally considered as an important risk factor in the occurrence of medication errors.

Considering unspecified requirements for completing prescription, the results of Palmero et al. (44.2% of the cases) were consistent to those of the present study (38% of the cases). In the present study, the absence of the name and /or signature of the physician and the illegibility of the medication order were 0.1 and 0.2%, respectively, but in the study of Palmero, they were 55 and 8.5%, respectively, which is inconsistent with our results [17, 19] .

Antibiotics are widely used drugs in NICUs. Accordingly, antibiotics mostly cause this prescribing error and studies of Esqué Ruiz et al. and Pawluk et al. have also confirmed this fact [17, 19]. We found that antibiotics were the most frequent (65%) medications involved in errors, too. The highly use of antibiotics in neonates increases the risk of necrotizing enterocolitis, hospital infection, prolonged hospitalization and, ultimately death.

The incidence of medication errors in the present study was similar to that of other studies. On the other hand, the frequency of prescribing errors in the present study was slightly less than that in other studies. The high rate of failing to administer the medication by nurse was another important point showing the difference.

In our region, many clinical departments of hospitals (especially ICUs) face insufficient number of beds (with a occupancy rate of 100% or more in most days of the year), nursing personnel, and manually written medication orders; these conditions cause a greater incidence of medication errors. Considering these aforementioned conditions, the measures such as using the Computerized Physician Order Entry System (CPOE), increasing the number of assistants in duty hours, increasing the number of NICU nurses based on international standards, reducing the number of shifts for a specific individual and availing drug therapy protocols for common diseases should be taken to reduce errors.

The limitations of this study included the impossibility of identifying and estimating the errors that need to be observed such as the rate of administration, insufficient knowledge about possible side effects of these errors on the neonates or removing them before administering.

## Conclusion

The present study showed that medication errors happen almost frequently in our NICUs. The most frequent medication errors were wrong dosage prescribed by physician in prescription phase, not administering the medication by nurse in administration phase, and on the quality of prescribing, lack of time and/or date of order. Overall medication errors happened more commonly in preterm neonates. The results of this study may help raise awareness of health professionals about the need for reducing medication errors in neonatal intensive care units.

## Data Availability

Available upon request from corresponding author.

## Abbreviations

CPOE: Computerized physician order entry system
NCCMERP: National coordinating council for medication error reporting prevention
NICUs: Neonatal intensive care units
